# Hydrogen Generation from Additive-Free Formic Acid Decomposition Under Mild Conditions by Pd/C: Experimental and DFT Studies

**DOI:** 10.1007/s11244-018-0894-5

**Published:** 2018-01-25

**Authors:** Felipe Sanchez, Davide Motta, Alberto Roldan, Ceri Hammond, Alberto Villa, Nikolaos Dimitratos

**Affiliations:** 1 Cardiff Catalysis Institute, School of Chemistry, Cardiff University, Main Building, Park Place, Cardiff, CF10 3AT UK; 2 Dipartimento di Chimica, Universitá degli Studi di Milano, via Golgi, 20133 Milan, Italy

**Keywords:** H_2_ production, Formic acid decomposition, Green chemistry, Renewable feedstock, Pd nanoparticles, DFT calculations

## Abstract

Safe and efficient hydrogen generation and storage has received much attention in recent years. Herein, a commercial 5 wt% Pd/C catalyst has been investigated for the catalytic, additive-free decomposition of formic acid at mild conditions, and the experimental parameters affecting the process systematically have been investigated and optimised. The 5 wt% Pd/C catalyst exhibited a remarkable 99.9% H_2_ selectivity and a high catalytic activity (TOF = 1136 h^−1^) at 30 °C toward the selective dehydrogenation of formic acid to H_2_ and CO_2_. The present commercial catalyst demonstrates to be a promising candidate for the efficient in-situ hydrogen generation at mild conditions possibiliting practical applications of formic acid systems on fuel cells. Finally DFT studies have been carried out to provide insights into the reactivity and decomposition of formic acid along with the two-reaction pathways on the Pd (111) surface.

**Electronic supplementary material:**

The online version of this article (10.1007/s11244-018-0894-5) contains supplementary material, which is available to authorized users.

## Introduction

Energy consumption around the world is increasing every day, which requires higher energy generation capacity, better energy management, and a shift away from non-renewable fossil fuels. The installation of a sustainable, secure and diversified energy supply chain is one of the greatest challenges to be addressed in this century.

Nowadays, industry and governments are increasing their attention on hydrogen as a candidate for clean energy due to the fact that its oxidation is highly exothermic and the only by-product is water [1]. Despite the potential of the use of hydrogen, its widespread utilization is currently limited by the capacity limitations of hydrogen storage technologies, and by the safety issues related with its storage and transportation under mild conditions [2].

Hydrogen gas is highly flammable in presence of oxygen and its traditional storage methods use compressed gas cylinders with pressure ranges between 200 and 350 bar. These high pressures require energy intensive processing and have safety risks, which makes public acceptance difficult, besides of the significant weight and volume requirements. During the last two decades, great scientific effort has been made in order to solve this problem, for instance, current research is exploring new methods to store or produce hydrogen under more secured and favourable conditions. These new methods can be classified depending on the interaction between hydrogen and the material, i.e. physisorbed or chemically incorporated in the structure. In the former method, hydrogen is adsorbed into a porous network such as zeolites [3], MOFs [4], clathrate hydrates [5], various carbon materials [3] and conventional organic polymers [6]. In the latter, a hydrogen-rich material is subjected to a decomposition process, which can be potentially reversible. Examples of these structures include solid phase systems, such as metal and non-metal hydrides [7], amines [8], amides [9], ammonia-like complexes [10], and liquid carriers such as *N*-ethylperhydrocarbazole [11], alcohols [12] or formic acid.

Catalytic hydrolysis of sodium and lithium borohydrides as well as ammonia borane have been widely studied because it provides a safe and low cost route to the production of hydrogen [10–13]. Ammonia decomposition has been also studied at temperatures below 500 K showing a significant reduction in activation energy when using carbon nanotubes catalysts promoted with cesium [14]. Metal hydrides such those of Mg–Al–Fe has been reported to achieve a maximum rate of 499.5 ml min^−1^ g^−1^ of hydrogen at 25 °C for Mg_60_–Al_30_–Fe_10_ (wt%) in 0.6 mol l^−1^ NaCl solution [15]. One of the most promising solutions consists on the utilization of hydrous hydrazine as reagent [16]. It presents the unique advantage that N_2_ is the only by-product for the complete decomposition [17].

Recently, formic acid, a major product formed during biomass processing, has been suggested and studied as a safe and convenient hydrogen storage material. It includes high volumetric hydrogen content (4.4 wt% of hydrogen) besides being liquid state at room temperature (volumetric capacity of 53.4 g l^−1^ at standard temperature and pressure), highly stable, environmental benign and nontoxic [18]. Furthermore, formic acid decomposition produces mainly gaseous products (H_2_/CO_2_) by decomposition. According to the U.S. Department of Energy, formic acid is one of the most promising hydrogen storage materials and its volumetric capacity surpasses that of most other storage materials today [19]. Hence, an effective and controlled release of hydrogen via selective decomposition of formic acid to CO_2_ and H_2_ is a desirable approach. More importantly, if the production of formic acid can be carried out under mild conditions via biomass conversion, a carbon neutral hydrogen storage cycle can be completed [20]. The proposed cycle can be closed when CO_2_ evolved during dehydrogenation of formic acid is reduced with an external supply of low purity H_2_ [21]. Formic acid decomposition occurs by two different pathways: dehydrogenation (1) and dehydration (2). Selective dehydrogenation is indispensable for the production of ultrapure H_2_ without undesirable dehydration, which also generates CO contaminants reducing the activity of Pd catalysts.

1 $${\text{Dehydrogenation: HCOOH}} \to {\text{C}}{{\text{O}}_2}+{{\text{H}}_2}\,\Delta {\text{G}}= - 48.4\,{\text{kJ\,mol}}^{ - 1}$$

2 $${\text{Dehydration: HCOOH}} \to {\text{CO}}+{{\text{H}}_2}{\text{O}}\,\Delta {\text{G}}= - 28.5\,{\text{kJ\,mol}}^{ - 1}$$

Previous studies have reported the utilisation of homogeneous catalysts to decompose formic acid at ambient temperatures and pressures. They showed promising results in terms of catalysts stability and selectivity to H_2_ and CO_2_ while significantly improving the catalytic efficiency [22–25]. However, the catalysts separation from the reaction mixture, moderate selectivity, their need for organic solvents/additives and, in several cases, harsh reaction conditions [26, 27], prevent them from scaling-up for practical applications. An alternative and attractive approach is the utilisation of heterogeneous catalysts that can achieve high catalytic activity [high turnover frequency (TOF) and utilisation of high substrate to metal molar ratio] at low temperature and with high selectivity towards H_2_ [19, 28].

One of the first reported studies on the formic acid decomposition were published in 1957 using Pd–Au alloy wires as catalytic materials [29] followed by studies using Pd/C. However, one of the main drawbacks was that the synthesised Pd/C catalysts deactivated quickly due to the poisoning intermediates resulting in its failing to applications [30]. Recent research has shown how to overcome this challenge by using a solution of formic acid and sodium formate of 9:1 volumetric ratio respectively and Pd/C reaching a TOF of 228.3 h^−1^ at 30 °C after 2 h [31]. Using this solution on a combination of Ag–Pd nanoparticles deposited on a basic resin a higher TOF of 820 h^−1^ at 75 °C was achieved with volumetric ratio of formic acid:sodium formate of 9:1 [32].

It has been reported that bimetallic nanoparticles can enhance the catalytic activity and selectivity compared to monometallic species. Recent studies by Xing and co-workers have shown the development of Pd–Au and Pd–Ag alloys supported on carbon to overcome the poisoning and stability issued on monometallic Pd analogues. These bimetallic particles generated high purity hydrogen production from the decomposition of formic acid at low temperatures. The authors also reported that the activities of Pd–Au/C and Pd–Ag/C can be enhanced by co-deposition with CeO_2_ [33]. In recent years, Tedsree et al. [34] developed a Ag–Pd core–shell catalyst supported on carbon based materials for dehydrogenation of formic acid resulting in a TOF of 626 h^−1^ although the drawback of generating CO due to the high temperature could not be avoided. A very recent research has reported that a metal–organic framework loaded with Ag–Pd alloy resulted in 100% selectivity for hydrogen generation from formic acid solution with TOF of 848 h^−1^ at 80 °C [35]. A wide variety of bimetallic and trimetallic Pd-based catalysts have been recently reported, e.g. AuPd [36, 37], PdNi [38], PdCo [39], PdCu [40], AuPdAg [41], CoAgPd [42] and NiAuPd [43] nanoparticles showing that the enhancement in the catalytic performance is mainly due to electronic and geometric effects.

The last years, research efforts led to improved experimental conditions, although for practical applications in portable electric devices, there are still limitations on component cost, catalyst deactivation, regeneration of by-products and control of the reaction kinetics, which current research tried to overcome.

Supported metal nanoparticles are important owing to their unique physical and chemical properties and various methods of preparation. Those methods have been investigated to synthesise metal nanoparticles with tailored size, shape and composition followed by their assembly and activation on support materials helping us to identify and minimise main drawbacks of traditional synthetic methodologies [44–49].

In the present work, we report the catalytic performance of an efficient commercial 5 wt% Pd/C for the production of hydrogen from the catalytic aqueous additive-free formic acid decomposition. 5 wt% Pd/C has been selected as a commercial reference and starting point for future research and optimisation of reaction conditions. The characterisation of these catalysts series (fresh and used) was thoroughly investigated by means of X-ray diffraction (XRD), X-ray photoelectron spectroscopy (XPS), transmission electron microscopy (TEM), scanning electron microscopy (SEM) with energy dispersive X-ray (EDX) and Brunauer–Emmett–Teller (BET) surface area analysis. The performance of the catalyst toward aqueous formic acid decomposition was carried out systematically in a batch reactor by varying a set of reaction parameters, such as substrate/metal molar ratio, stirrer speed, temperature and concentration of formic acid. Kinetic isotope studies and reusability tests were as well performed. Finally, periodic density functional theory (DFT) calculations were employed to gain insights on the energetics of formic acid decomposition on Pd (111) surface as the most represented model.

## Experimental and Computational Methodologies

### Materials and Chemicals

5 wt% Pd/C was purchased from Sigma-Aldrich (Cat. 20, 568-0, 5 wt% Palladium on activated carbon). Formic acid (98%) was obtained from Fischer Scientific. Succinic acid (99%) from Sigma-Aldrich (Cat. S3674-100G). For the kinetic isotope studies, HCOOD (95% in D_2_O), DCOOH (95% in H_2_O), and DCOOD (95% in D_2_O) were purchased from Sigma-Aldrich. Deionised water was used as reaction solvent.

### Catalyst Characterisation

XRD data were collected at ambient temperature with PANanalytical X’PertPRO X-ray diffractometer using Cu Kα radiation and operated at 40 kV and 30 mA. XRD patterns were recorded between 10°–80° 2θ at a step size of 0.017°. X-ray photoelectron spectra (XPS) was recorded on a Kratos Axis Ultra DLD spectrometer using a monochromatic Al Kα X-ray source. X-ray source (75–150 W) and analyser pass energies of 160 eV (for survey scans) or 40 eV (for detailed scans). Samples for examination by TEM were prepared by dispersing the catalyst powder in high purity ethanol using ultra-sonication. 40 µl of the suspension was dropped on to a holey carbon film supported by a 300 mesh copper TEM grid before the solvent was evaporated. The samples for TEM were then examined using a JEOL JEM 2100 TEM model operating at 200 kV. The morphology and composition was examined by scanning electron microscope (SEM) Hitachi TM3030PLUS equipped with a Quantax70 energy-dispersive X-ray spectroscope (EDX). BET measurements were carried out at liquid nitrogen atmosphere using a Quantachrome Autosorb equipment. After outgassing the samples, porosimetry measurements were performed (120 °C, 3 h). Surface area was calculated using the BET method based on adsorption data in the partial pressure (P/P_o_) range 0.05–0.35. Pore volume was calculated by a single point method from the amount of nitrogen adsorbed at P/P_o_ = 0.99. Average pore size has been calculated from the desorption branch of the isotherm. Microwave Plasma analysis of the filtrated solutions after reaction were performed using a Microwave Plasma Atomic Emission Spectroscopy instrument (4100 MP-AES) to investigate the possibility of Pd leaching from the catalyst.

### Formic Acid Decomposition and Analytical Methods

Liquid-phase formic acid decomposition was performed in a two-necked 100 ml round-bottom flask, which was placed in an oil bath with a reflux condenser and a magnetic stirrer at a pre-set temperature (30–60 °C). Typically, 10 ml of the desired concentration (0.5 M) of HCOOH aqueous solution was placed into the reactor. Once, the solution reached the target temperature, the desired amount of catalyst was added and the reaction was initiated by stirring. Each reaction was performed at least twice or three times in some cases so as to check the reproducibility of the data. In order to compare different reactions TOF (amount of reactant converted per mole of metal per time) has been calculated for the first 5 min.

#### Product Analysis

Formic acid conversion was calculated using HPLC (high-performance liquid chromatography). Liquid samples of the reaction mixture were withdrawn periodically (0.1 ml), diluted to 10 ml using deionised water and analysed by HPLC, Agilent 1220 Infinity LC using a column Agilent MetaCarb 87H 250 × 4.6 mm at 60 °C equipped with an variable wavelength detector (VWD) at 210 nm. The eluent was an aqueous solution of phosphoric acid (0.1 wt%) and the flow rate was set to 0.4 ml min^−1^. Succinic acid was used as an external standard for the quantification of formic acid (Fig. S1A).

#### Gas Analysis

The volume of the evolved gas was monitored by recording the displacement of water in a gas burette. Analysis for the detection of H_2_ and CO_2_ was performed using a QGA-MS from Hiden Analytical. CO was quantified and CO_2_ content double-checked using a Varian 450-GC fitted with a CP-Sil 5CB capillary column (50 m length, 0.32 mm diameter, carrier gas: He), a methanator unit and both FID and TCD detectors with a detection limit of CO below 5 ppm. The gases were quantified using calibration curves (Fig. S1B) constructed from commercial standards (BOC gases). Between 4 and 5 ppm of CO were found and between 40,000 and 50,000 ppm of CO_2_ (Fig. S2), which means that the reaction mainly follows the desired route (to hydrogen and carbon dioxide). The H_2_/CO_2_ ratios were evolved from 1.06 to 1.13 for the catalytic decomposition of formic acid. This slight deviation could be due to a difference in solubility of CO_2_ and H_2_ or a consumption and adsorption of H_2_ by PdO as previously reported [50].

#### Recyclability Tests

The recyclability tests were performed at 30 °C using a formic acid concentration of 0.5 M HCOOH. After the completion of the liquid-phase decomposition of formic acid, the catalyst was isolated from the reaction solution by filtration and then dried at ambient temperature for 18 h. The dried catalyst was used again in the catalytic decomposition of the aqueous solution of formic acid. The recycling tests were repeated for five times. After the second cycle of reusability, the gas produced was also analysed so as to compare with the gas produced by the fresh catalyst. This experiment shows H_2_/CO_2_ volumetric ratios of 0.84–1.04.

#### Order of Reaction and Kinetic Isotope Effects (KIE)

In order to calculate an approximation of the reaction order, knowing the amount of gas produced and so, the initial rate, (the total gas volume of CO_2_ and H_2_ produced within the first 5 min), the reaction order can be estimated representing the rate vs. the concentration of formic acid and fitting to a power-law model equation:

$$r=k \times {C^n}$$

where r is the reaction rate, k is the kinetic coefficient, C is the initial formic acid concentration and n is the reaction order.

KIE were carried out for elucidating reaction pathways using the three isotopes of HCOOH under the same experimental conditions of the reusability test.

### Computational Methods

Periodic plane-wave DFT calculations were performed using the Vienna ab-initio simulation package (VASP) [51, 52], the Perdew–Burke–Ernzerhof functional revised for solids [53] and a kinetic energy of 500 eV to expand the plane-waves of the Kohn–Sham valence states [54]. The inner electrons were represented by the projector-augmented wave (PAW) pseudopotentials considering also non-spherical contributions from the gradient corrections [55]. All the calculations include the long-range dispersion correction approach by Grimme [56, 57], which is an improvement on pure DFT when considering large polarizable atoms [58–63]. We included a self-consistent aqueous implicit solvation model [64, 65]. The optimisation thresholds were 10^−5^ eV and 0.03 eV/Å for electronic and ionic relaxation, respectively. The Brillouin zone was sampled by Γ-centred k-point mesh generated through a Monkhorst–Pack grid of 5 × 5 × 1 k-points, which ensures the electronic and ionic convergence [66]. In order to improve the convergence of the Brillouin-zone integrations, the partial occupancies were determined using the first order Methfessel–Paxton method corrections smearing with a set width for all calculations of 0.2 eV. Open shell calculations were tested leading to close shell results.

The Pd bulk lattice parameter is 3.893 Å [67] which is in very good agreement to the one resulting from the cell optimization (3.836 Å). The (111) surface is simulated by a slab model containing five atomic layers where the two uppermost layers were relaxed without symmetry restrictions and the bottom ones were frozen at the bulk lattice parameter. The slab contains 45 atoms per unit cell exposing an area of 66.210 Å^2^. We added a vacuum width of 15 Å between periodic slabs, big enough to avoid the interaction between periodic images.

We defined the binding energy (E_B_) as the difference between the isolated species and the combined system and the reaction energy (E_R_) of each step is calculated as the total energy difference between the final state (product(s)) and the initial state (reactant(s)).

## Results and Discussion

### Catalyst Characterisation

**Fig. 1:**
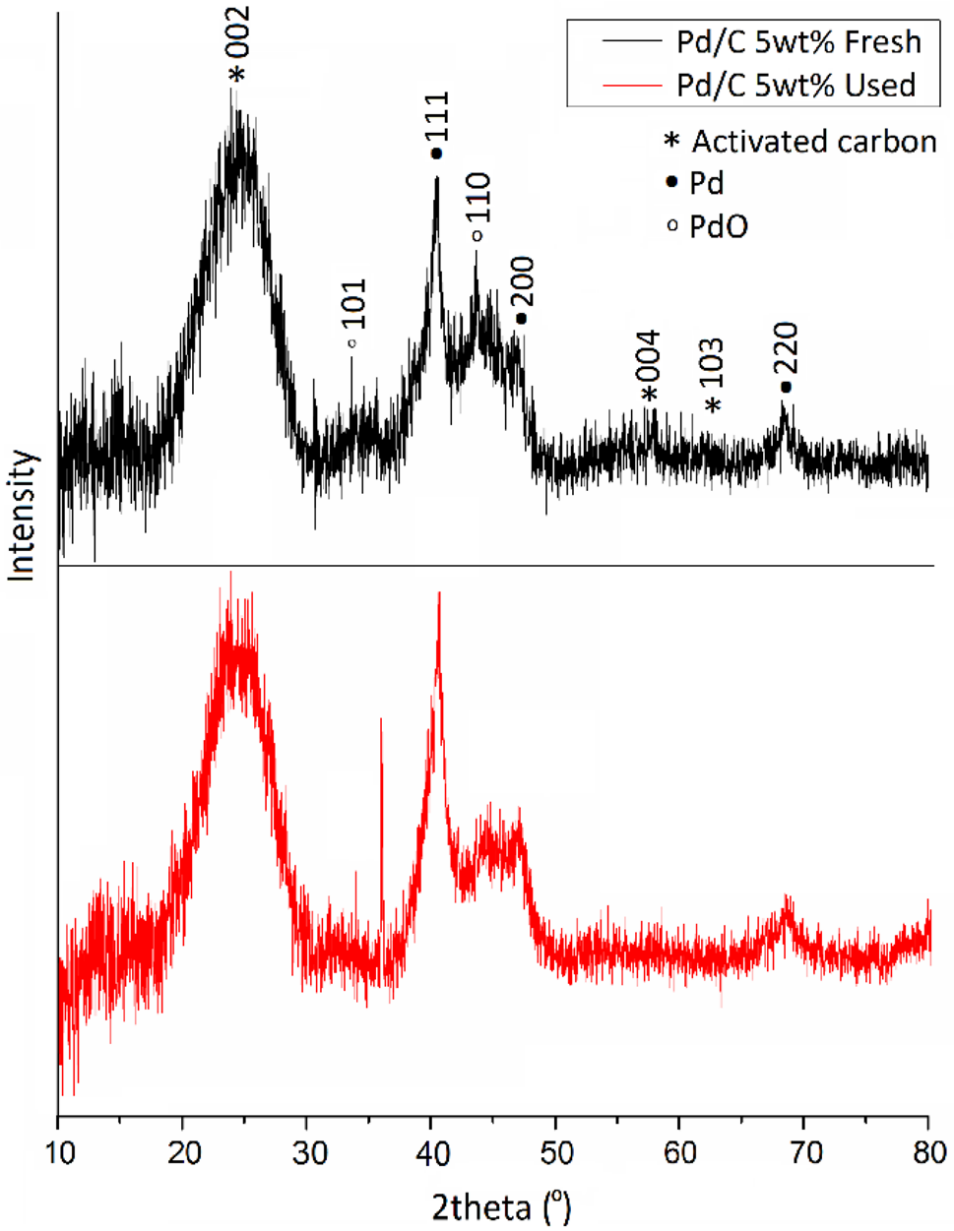
XRD patterns of fresh and used 5 wt% Pd/C catalyst

XRD patterns of the fresh and used catalysts are shown in Fig. 1. The broad diffraction peak at 2θ = 24.7° and the peaks at 57.3° and 61.8° are related to the carbon support, in particular, to the (002), (004) and (103) planes of the activated carbon respectively [50]. The diffraction peaks at 2θ = 40.4°, 46.8° and 68.3° correspond respectively to the (111), (200) and (220) characteristic planes of face-centered cubic structure of Pd. The peaks at 35.8° and 43.8° correspond to PdO (101) and (110) respectively [68]. Characterisation of the used catalyst (Fig. 1) indicates the decrease of the intensity of the diffraction peaks at 2θ = 35.5° and 43.8° and the increment of the diffraction peaks at 2θ = 40.4° ascribed to (111) of Pd due to the reduction of PdO to metallic Pd from the in situ H_2_ generation during reaction progress. This difference has been further investigated by means of XPS studies.

**Fig. 2:**
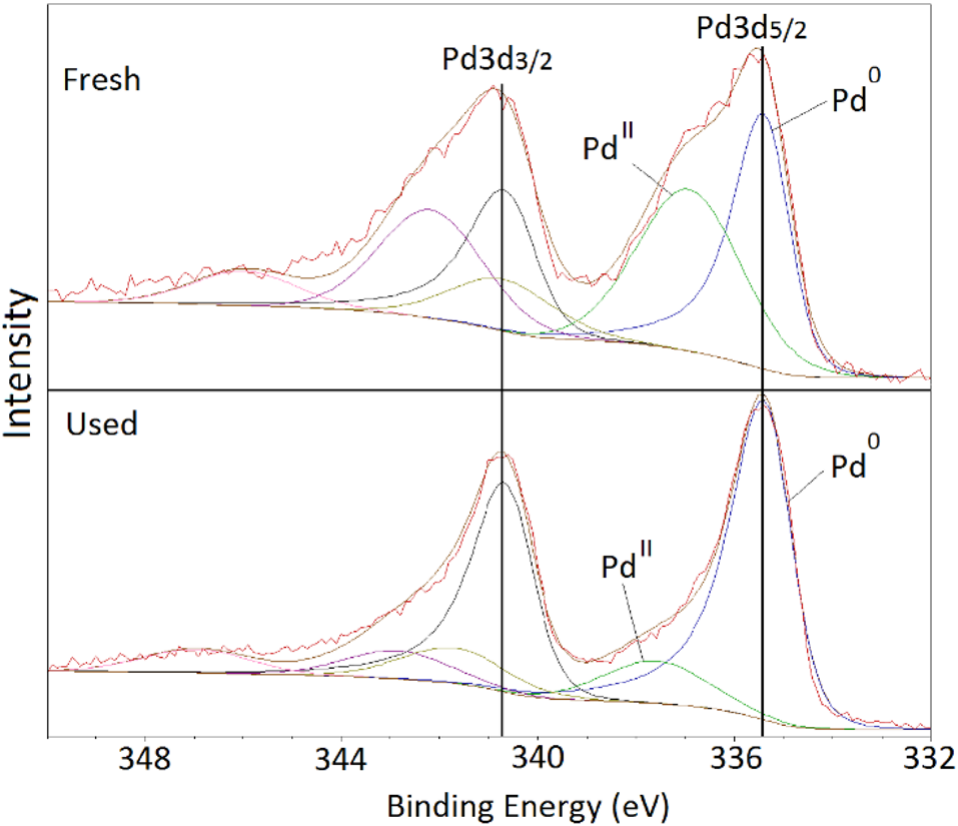
XPS spectra of fresh and used 5 wt% Pd/C catalyst

XPS analysis of the Pd(3d) region is presented in Fig. 2. Each Pd species displayed two peaks due to the Pd 3d_5/2_ and Pd 3d_3/2_ transitions. For the fresh catalyst, the peaks at 335.4 and 340.7 eV correspond to Pd 3d_5/2_ and Pd 3d_3/2_ transitions respectively and are assigned to the presence of Pd^0^. The peaks at 337.0 and 342.3 eV correspond to Pd^II^ being PdO the most probable species [31]. Chlorine impurities have been studied since it can act as catalysts poison. A negligible Cl content of 0.1 and 0.05% was found for the fresh and used catalysts. For the fresh catalyst, the ratio Pd^0^/Pd^II^ was approximately 0.41 whereas for the used catalyst, it increased to 0.71. The decrease of the intensity of the peaks assigned to Pd^II^ and the simultaneous increase of Pd^0^ for the used catalyst are consistent with the data obtained from XRD for the reduction of Pd^II^ to metallic Pd.

**Fig. 3:**
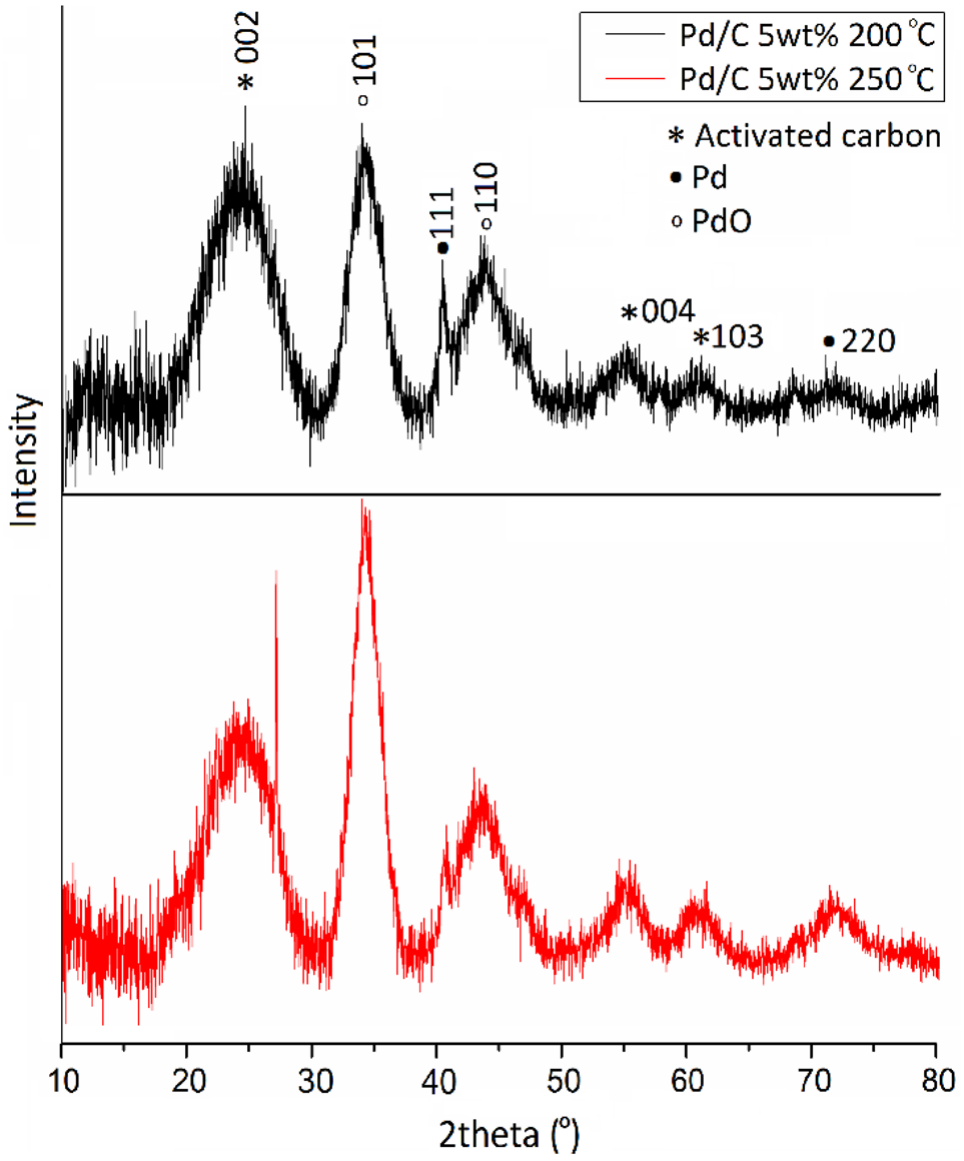
XRD patterns of 5 wt% Pd/C catalyst calcined at 200 and 250 °C

**Fig. 4:**
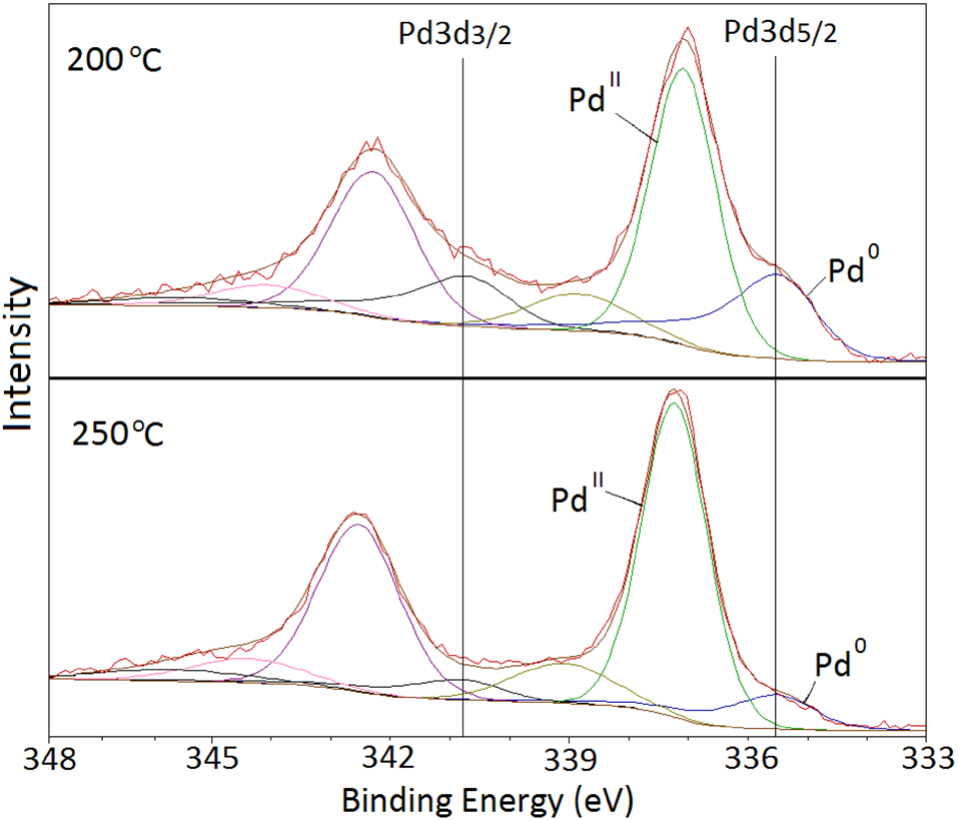
XPS spectra of 5 wt% Pd/C catalyst calcined at 200 and 250 °C

In order to get insights on the effect of thermal treatment, fresh Pd/C catalyst was calcined at 200 and 250 °C in flowing air for 3 h. This range of temperature is selected to study the impact of heat treatment on the (i) catalytic activity, (ii) oxidation state and particle size of Pd and (iii) maintaining the thermal stability of the activated carbon support. XRD patterns are shown in Fig. 3. The diffraction peaks assigned to PdO increased after the heat treatment and a significant decrease of the diffraction peak corresponding to metallic Pd was observed when compared with the fresh catalyst (Fig. 1). These changes indicate, as expected, the facile oxidation of metallic Pd to Pd^II^ since metallic Pd tends to readily oxidise when exposed to air at high temperatures in agreement with XPS data. The decrease of the diffraction peak at 2θ = 40.4° supports this observation. When increasing the calcination temperature to 250 °C this reduction of the intensity of the diffraction peak was more noteworthy due to the lesser presence of metallic Pd in the catalyst. XPS analysis of the calcined samples are shown in Fig. 4, which confirmed the decrease of metallic Pd and the progressive oxidation to PdO as the heat treatment temperature increased. From a molar ratio of Pd^0^/Pd^II^ of 0.41 in the fresh catalyst, it progressively decreased to 0.28 and 0.12 for the Pd/C treated at 200 and 250 °C respectively.

**Fig. 5:**
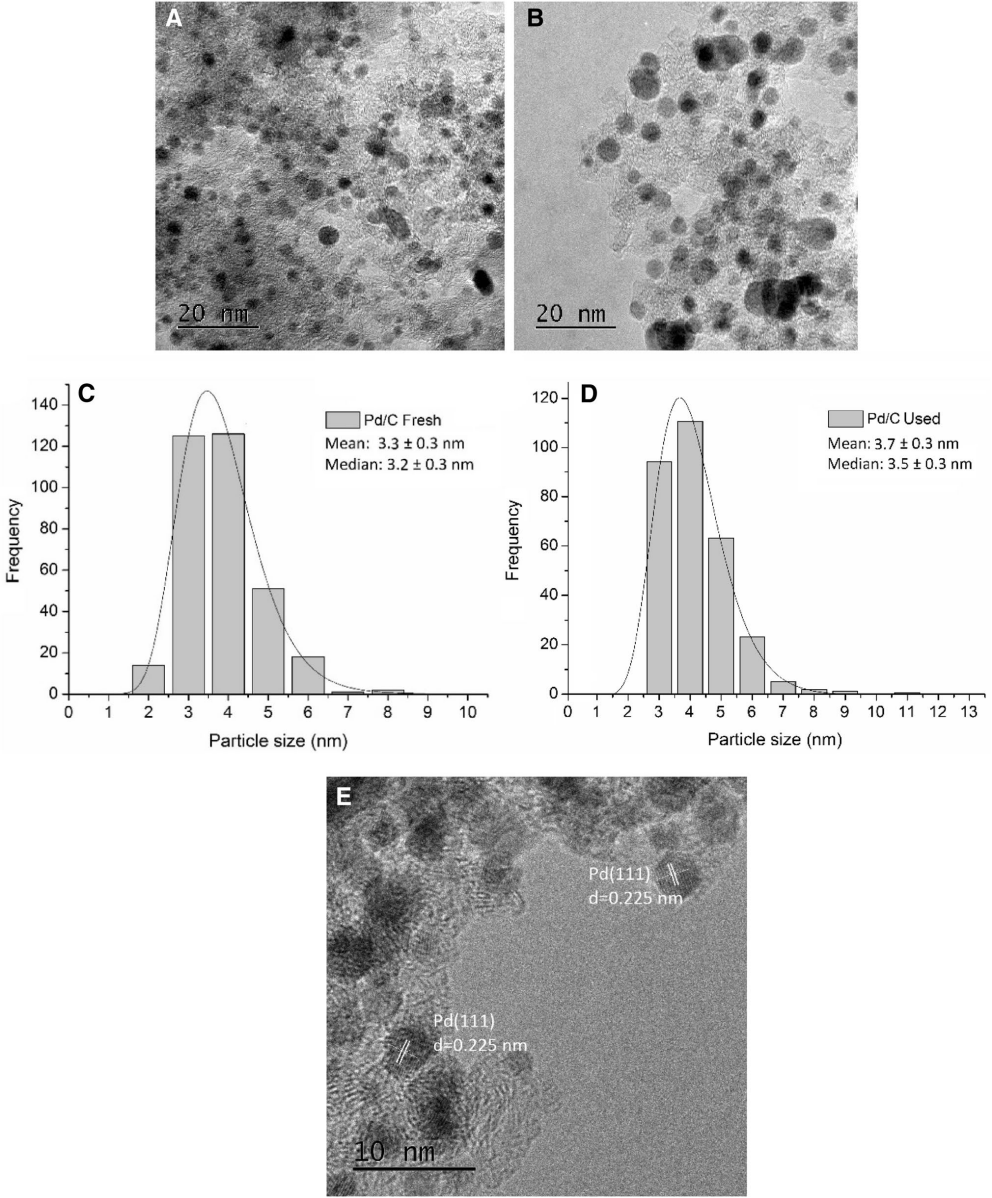
Characterisation of fresh and used 5 wt% Pd/C catalyst. Magnification: ×250k **a** TEM of the fresh 5 wt% Pd/C, **b** TEM of the used 5 wt% Pd/C. Particle size distribution **c** 5 wt% Pd/C fresh, **d** 5 wt% Pd/C used, **e** HRTEM image of the fresh 5 wt% Pd/C. Magnification: ×600k

TEM analysis was performed to determine the particle size distributions and mean particle size of the fresh and used Pd/C catalysts. TEM images are shown in Fig. 5 indicating a good dispersion of the Pd nanoparticles. The mean particle size distributions for the fresh and used catalysts were in the range 2–6 nm (Fig. 5c, d). The mean particle size of Pd nanoparticles slightly increased from 3.3 ± 0.3 nm to 3.7 ± 0.3 nm for the used. This slight increase of particle size, caused by agglomeration, could explain the small decrease in catalyst activity after the first use. The HRTEM image in Fig. 5e for the fresh commercial 5 wt% Pd/C showed a discrete lattice-fringe of the face centered cubic (fcc) Pd crystal with a d-spacing of 0.225 nm, which is in reasonable agreement with the lattice spacing of the (111) plane in our computational model (0.271 nm) taking into account the atomic radius of the element [69]. The TEM images of the calcined catalysts at 200 and 250 °C are displayed in Fig. S3A and S3B. These images show no apparent change of the mean particle size when Pd/C is calcined at 200 °C, however, at 250 °C the mean particle size increased to 4.0 ± 0.3 nm due to a low degree of sintering (Fig. 3SC and 3SD). The HRTEM images in Fig. S3E and S3F present lattice-fringes with a d-spacing of 0.225 nm for the catalyst calcined at 200 °C, in agreement with the fresh Pd/C catalyst, although at 250 °C, a certain number of particles showed an additional lattice fringe with d-spacing of 0.26 nm assigned to the PdO (002) or PdO (101) [70, 71].

**Fig. 6:**
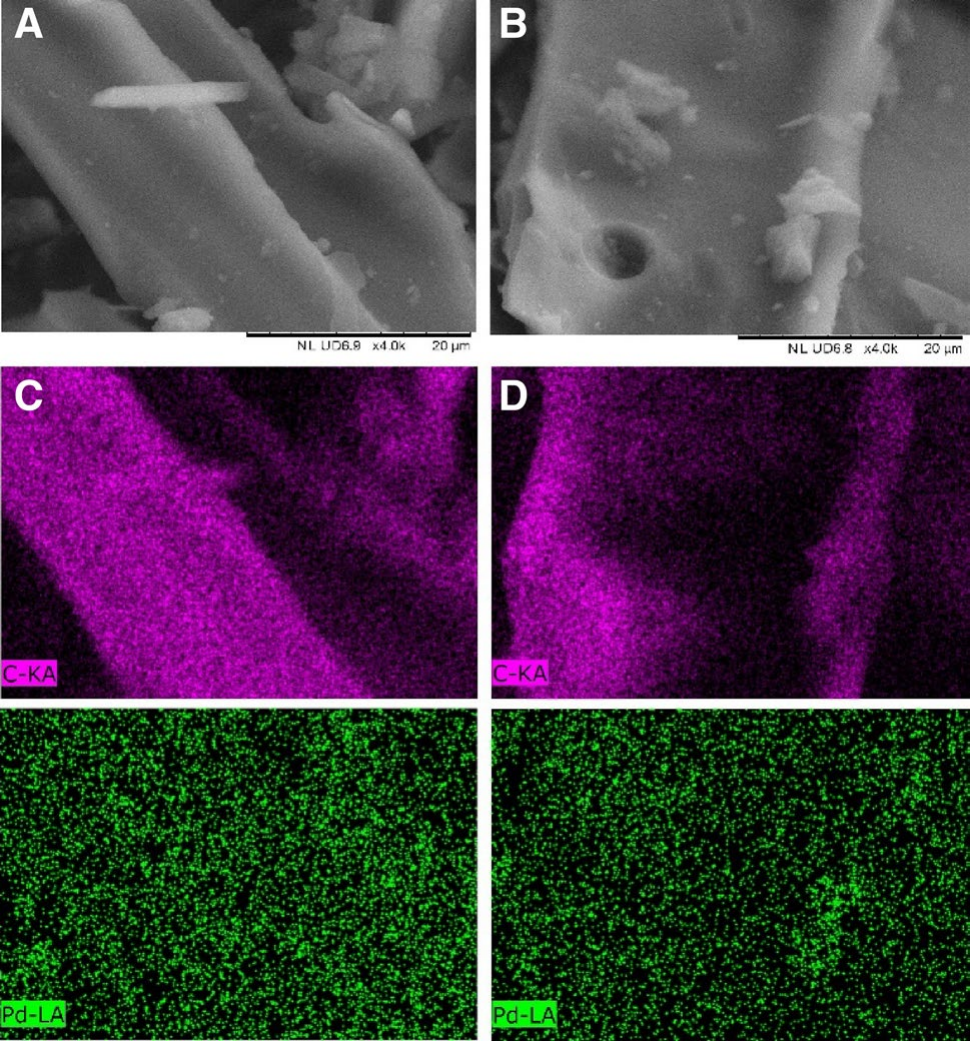
Characterisation of fresh and used 5 wt% Pd/C catalyst. **a** SEM of the fresh 5 wt% Pd/C, **b** SEM of the used 5 wt% Pd/C, **c** EDX of the fresh 5 wt% Pd/C, **d** EDX of the used 5 wt% Pd/C

The morphology and metal loading of the Pd/C catalyst was examined before and after the catalytic reaction by means of SEM–EDX. No significant changes are apparent in the morphology of the fresh and used catalyst. The composition of the Pd nanoparticles was analysed by means of EDX for the fresh and used catalyst. The EDX spectra shown in Fig. 6c, d confirm the theoretical value of 5% displaying an average 5.1 and 5.0 wt% of Pd for the fresh and used catalyst respectively. However, compared with the fresh catalyst, the used catalyst showed a lower degree of homogeneous Pd particles distribution and the formation of agglomerated particles is evident in some areas; these can have a negative impact on the catalyst activity during recycling tests.

BET surface area of the fresh Pd/C catalyst was evaluated and it was found to be 820 m^2^ g^−1^ with pore volume and pore size of 0.73 cc g^−1^ and 3.56 nm respectively, which, according to the IUPAC classification, is characteristic of solid with mesoporous (2–50 nm) structure [72]. For the used catalyst, a BET surface area of 800 m^2^ g^−1^ and pore volume and pore size of 0.77 cc g^−1^ and 3.86 nm respectively was obtained. We can conclude that this reaction does not overly affect negatively the porous structure of the catalyst.

### Formic Acid Decomposition

**Fig. 7:**
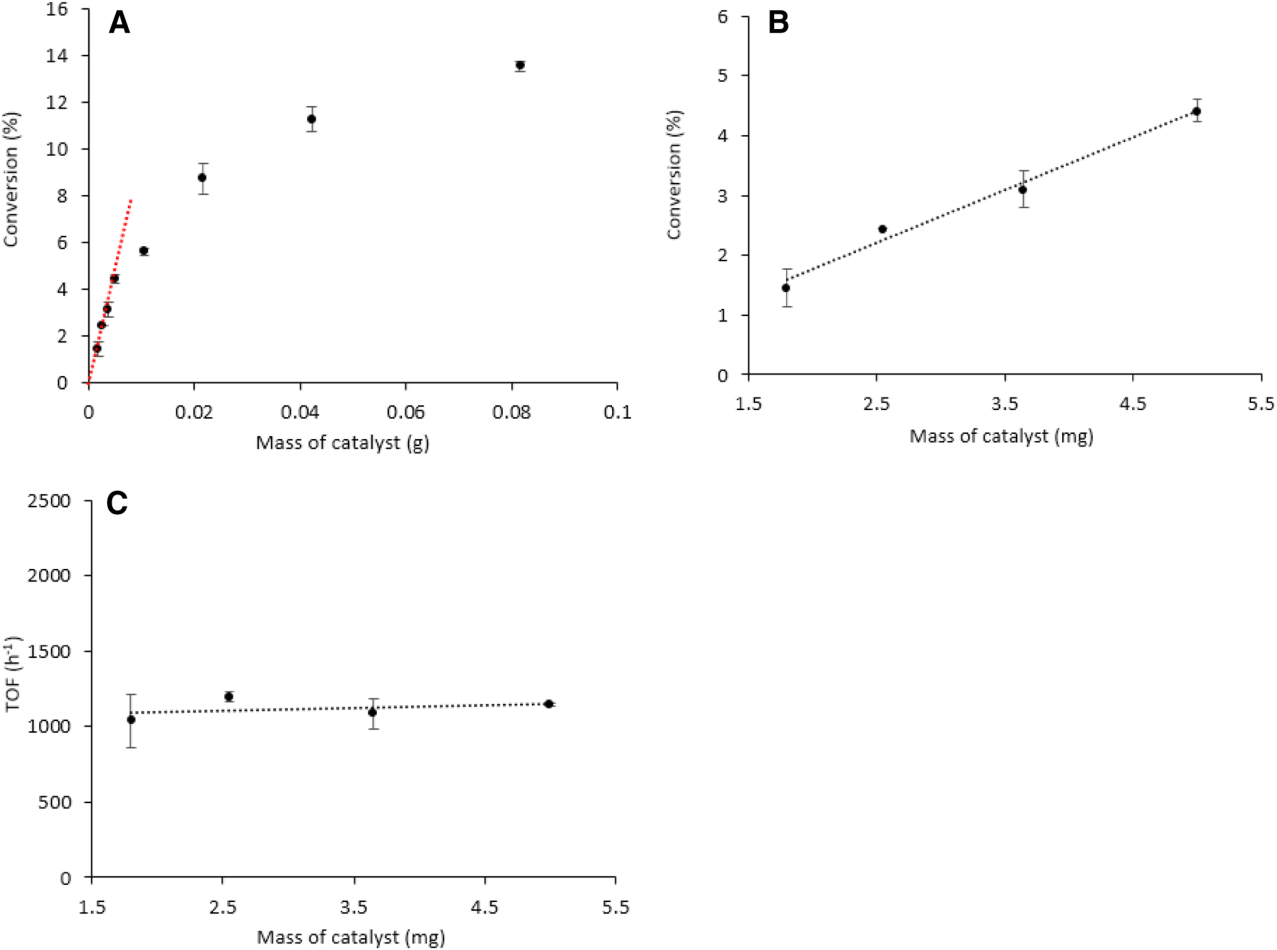
Effect of catalyst mass on **a** conversion, **b** conversion in a substrate/metal molar ratio range from 2000 to 6000 and **c** TOF. Reaction conditions: 30 °C, 0.5 M HCOOH, 750 rpm, 2 h reaction time

We investigated the effect of the reaction conditions on the commercial 5 wt% Pd/C for the decomposition of formic acid. Initially, the effect of formic acid/metal molar ratio (125–6000, equivalent to 81–1.8 mg of catalyst respectively) was studied at 30 °C, stirring rate of 750 rpm and for a reaction time of 2 h. We identified two reaction regimes as shown in Fig. 7a. In the range of substrate/metal molar ratio of 2000–6000 (5.3–1.8 mg), the red line in the Fig. 7a, the conversion is proportionally dependant with the amount of catalyst, which means that the reaction is in kinetic regime. This fact is more clearly presented in Fig. 7b. It agrees with the constant TOF values confirming that there are not diffusion limitations apparent under this regime as shown in Fig. 7c. Diffusion limitations are present when the catalyst mass is above 5.3 mg, Fig. 7a.

**Fig. 8:**
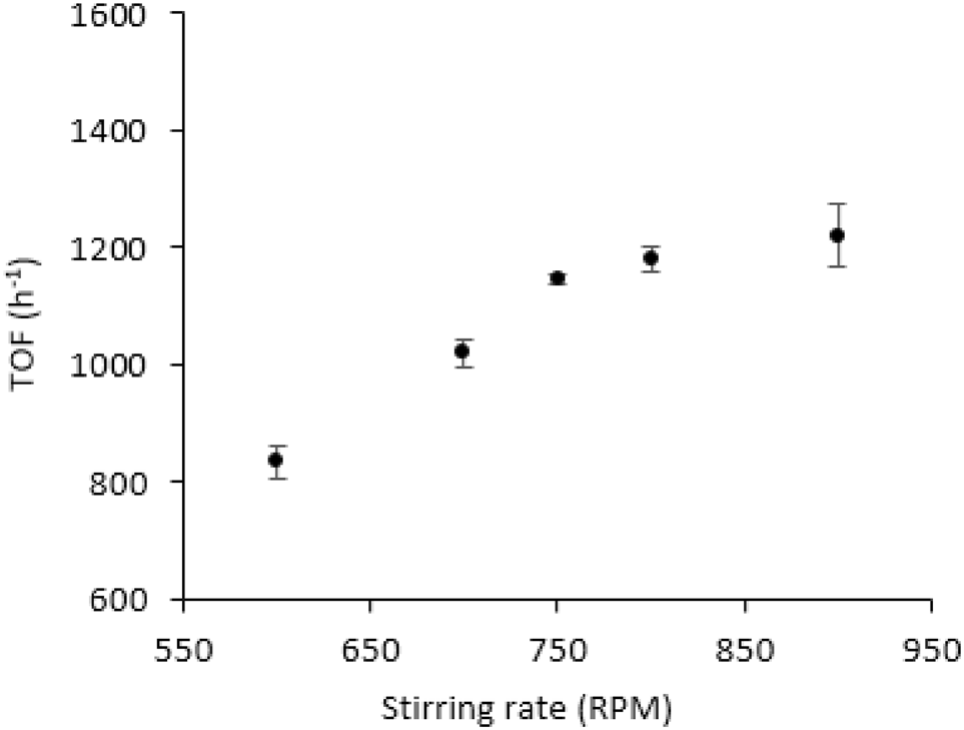
Effect of stirrer rate on TOF. Reaction conditions: 5.3 mg of catalyst (substrate/metal molar ratio: 2000:1), 30 °C, 0.5 M HCOOH, 2 h reaction time

We then investigated the effect of stirring rate, which varied in the range of 400–900 rpm at 30 °C and using substrate/metal molar ratio of 2000 (5.3 mg). Increasing the stirring rate will increase the reactants collision with the solid catalyst and consequently influence the rate of reaction as shown in Fig. 8. By increasing the stirring speed from 600 to 750 rpm, the TOF values showed a significant increment suggesting that the reaction is under diffusion regime. In the range 750–900 rpm, the TOF values were slightly increased and reached a plateau indicating that the stirrer speed at this range has a minor effect in the conversion and therefore, the reaction is in kinetic regime. Considering the above results, a stirrer rate of 750 rpm was selected as the optimum value for subsequent studies.

**Fig. 9:**
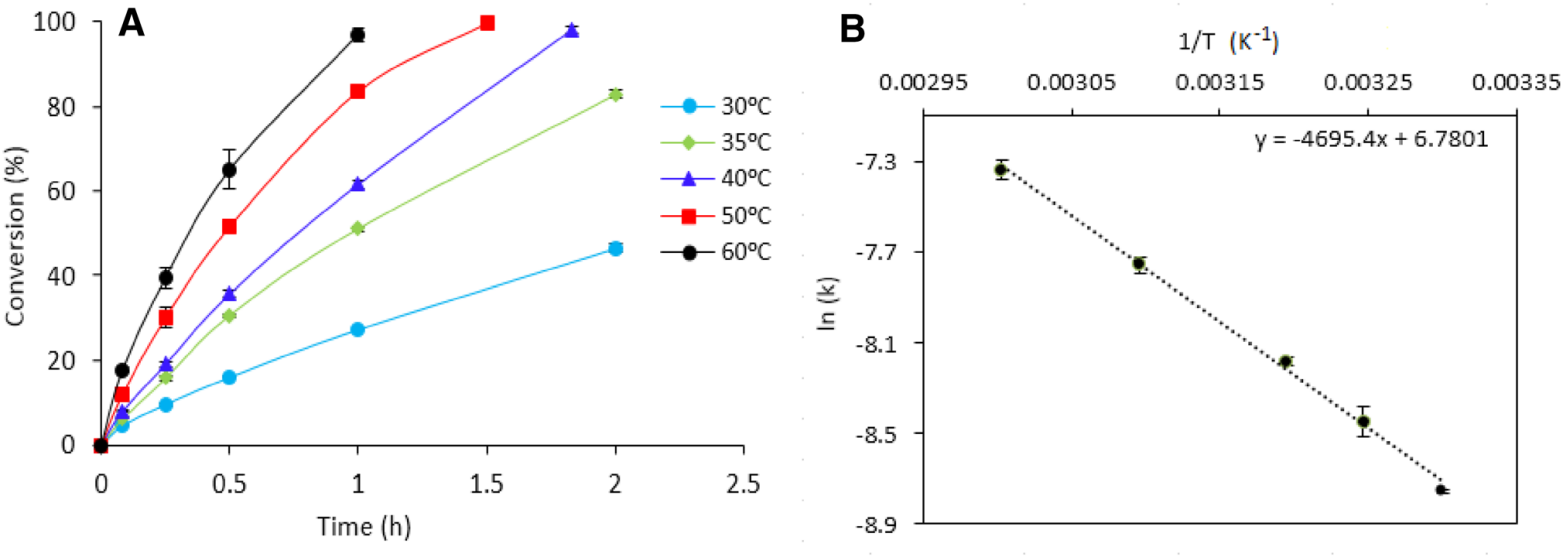
**a** Effect of temperature on conversion. **b** Arrhenius plot. Range 30–60 °C. Reaction conditions: 5.3 mg of catalyst (substrate/metal molar ratio: 2000:1), 0.5 M HCOOH, 750 rpm, 2 h reaction time

We further studied the effect of temperature on the formic acid decomposition in the range 30–60 °C at 750 rpm and a substrate/metal molar ratio of 2000, summarised in Fig. 9a. Higher temperatures were not investigated since one important requirement of portable devices utilising formic acid fuel cells is the necessity of working under mild conditions. Conversion of formic acid was enhanced with the increase of temperature as expected. The apparent activation energy $\left( {{\text{E}}_{{\text{a}}}^{{{\text{app}}}}} \right)$ for the reaction was calculated by the slope of the Arrhenius plot as shown in Fig. 9b with a value of 39 kJ mol^−1^. This value is one of the lowest values reported for a monometallic Pd catalyst under kinetic regime [50].

**Fig. 10:**
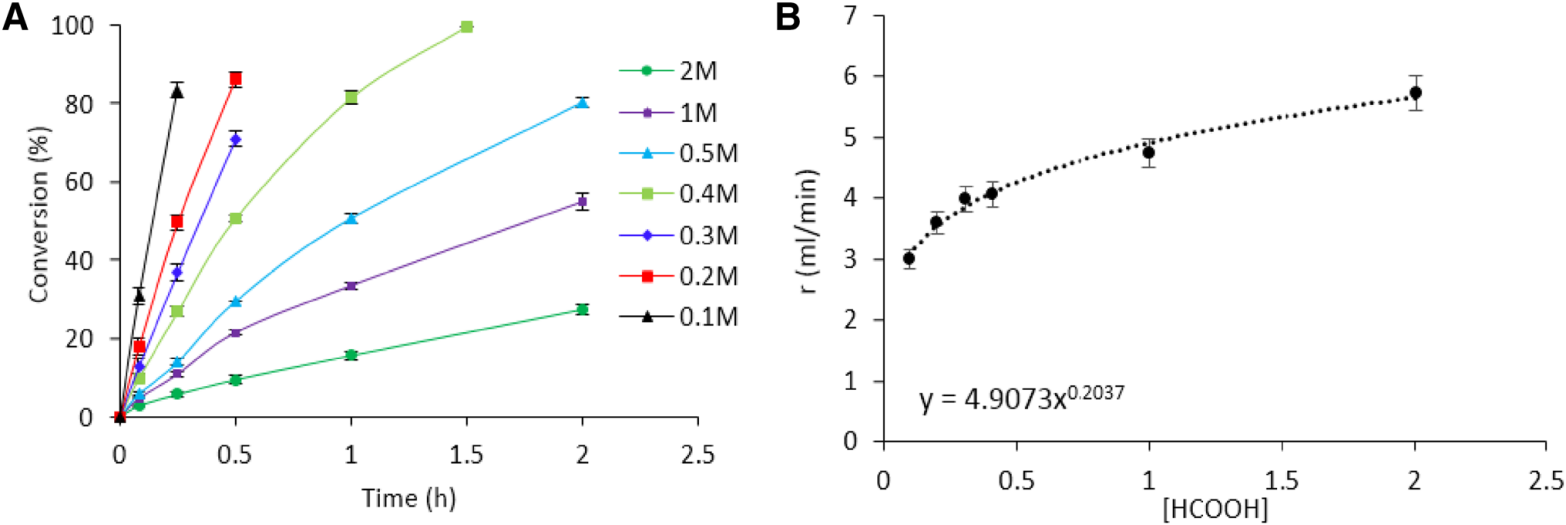
**a** Effect of concentration of formic acid. **b** Rate vs. [HCOOH]. Reaction conditions: 5.3 mg of catalyst (substrate/metal molar ratio: 2000:1), 30 °C, 750 rpm, 2 h reaction time

Another parameter investigated was the effect of formic acid concentration for determining the reaction order (Fig. 10a). A set of experimental reactions with a concentration range of formic acid from 0.1 to 2 M at 30 °C and substrate/metal molar ratio of 2000 were performed and the fitting to the power-law equation model in the rate vs. concentration plot led to a reaction order of 0.2 (Fig. 10b).

An initial TOF of 1136 h^−1^ was produced at the optimised conditions of substrate/metal molar ratio of 2000, 750 rpm, 30 °C and a concentration of formic acid of 0.5 M. Table 1 summarises the comparison between TOF and activation energy obtained in this study as well as previous reported values.

**Table 1:** Comparison of heterogeneous catalysts toward formic acid decomposition

Catalyst	T (°C)	Reagent	TOF (h^−1^)		Activation energy (KJ mol^−1^)	Ref.
			Initial	2 h		
Pd/C	30	Formic acid (0.5 M)	1136		39	This work
Au_41_Pd_59_/C	50	Formic acid (1 M)	230		28 ± 2	[73]
Ag@Pd (1:1)	35	Formic acid		156	30	[34]
Ag_42_Pd_58_	50	Formic acid (1 M)	382		22 ± 1	[74]
Ag_18_Pd_82_@ZIF-8	80	Formic acid (1.5 M)/sodium formate (0.5 M)	580		51.3	[4]
1 wt% Pt	100	Formic acid		32.4	51	[75]
10 wt% Pt	100	Formic acid		43	64	[75]
Pd/C	30	Formic acid (0.2 M)/sodium formate (1.8 M)		228.3	–	[31]
Pd/C	30	Formic acid (1.33 M)	48	28	53.7	[50]
PtRuBiO_x_	80	Formic acid (1.15 M)	312		37	[76]
Pd–Ag/C	92	Formic acid (9.94 M)/sodium formate (3.33 M)	22		115	[33]
Pd–Au/C	92		45		138.6	[33]

**Fig. 11:**
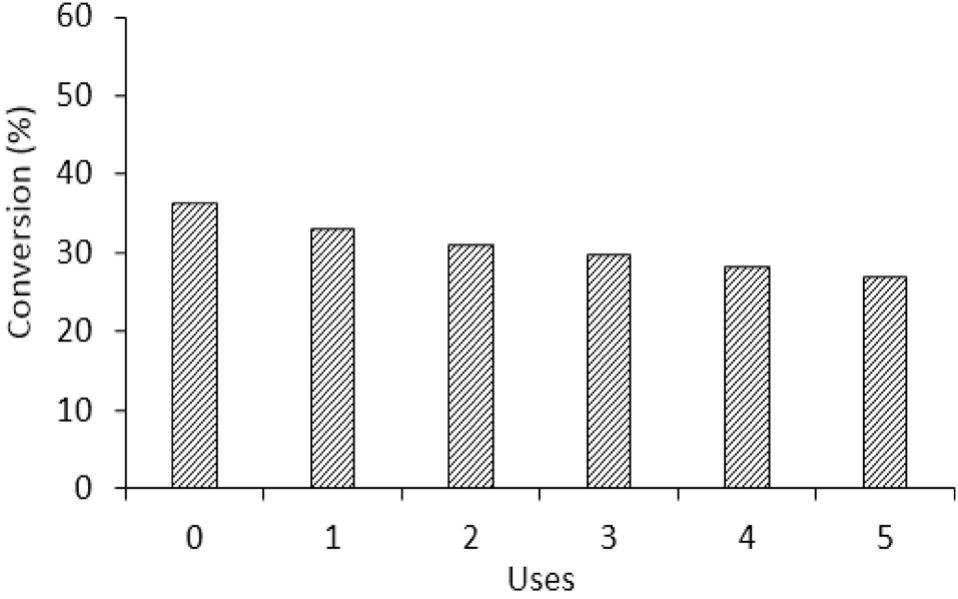
Reusability of 5 wt% Pd/C after five uses. Reaction conditions: 5.3 mg of catalyst (substrate/metal molar ratio: 2000:1), 30 °C, 750 rpm, 1.5 h reaction time

Durability and recyclability of a catalyst is a crucial aspect for practical applications. The stability of the commercial 5 wt% Pd/C catalyst was investigated at 30 °C for five subsequent catalytic cycles of 1.5 h each. As shown in Fig. 11, our analysis revealed that the Pd/C catalyst preserved 72% of its initial activity in its fifth use. This slight decrease in the activity can be due to several factors: strong absorption of formic acid on the surface of the catalyst, poisoning from CO, Pd nanoparticles agglomeration or decrease of Pd loading by leaching. MP-AES analysis of the filtered liquid after every use of the reusability test showed negligible Pd concentration (Table 2) below the detectability limit of the instrument (0.05 ppm being the initial amount of Pd 26.5 ppm). The gas analysis performed to the gas-phase products of the second cycle reaction showed as well a low CO evolution from 4 to 6 ppm which supports the promising results obtained during the reusability tests. Strong absorption of formic on the surface and agglomeration are the most probable reasons of the slight decrease in catalytic activity.

**Table 2:** MP-AES analysis of the filtered liquid

Use	Concentration of Pd (ppm)	% of Pd resulting
Fresh	0.06	0.22
1	0.04	0.15
2	0.04	0.15
3	0.04	0.15
4	0.07	0.26
5	0.04	0.15

**Scheme 1:**
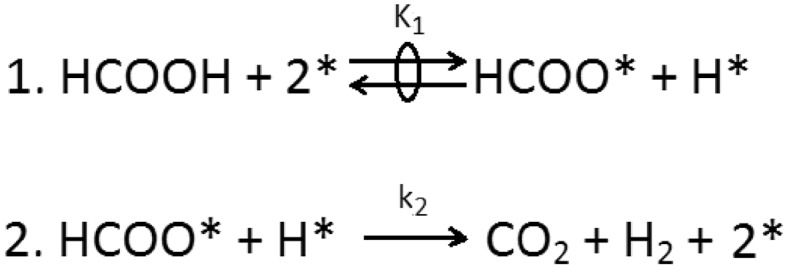
Possible pathway for formic acid decomposition

To gain additional insights about the rate-determining step of the reaction, KIE were calculated using HCOOD, DCOOH and DCOOD. KIE values are the ratio between the reaction rate when HCOOH is used and the reaction rate when an isotopomers is used. These values are presented in Table 3. In case of KIE values for HCOOD close to 1 but larger value for DCOOH, it means that HCOOH dissociation is kinetically relevant. On the contrary, KIE values close to 1 for DCOOH but not for HCOOD is due to limitation by the formate decomposition. Finally, in case of KIE values of approximately 1 for both HCOOD and DCOOH, it can be explained by a limiting hydrogen desorption both from HCOOD and DCOOH [77]. In our experiments, HCOOD provided the closest KIE values to unity while, both DCOOH and DCOOD presented larger values (Fig. S4). These results show that the rate determining step is the cleavage of the bond C–H/D, in agreement with the simulated thermodynamic profile explained bellow. As presented in Scheme 1, the most probable pathway for the formic acid decomposition is the formation of formates. Thus, the H-atoms formed after the O–H dissociation recombines with the H-atoms formed after the formate decomposition yielding CO_2_ and H_2_.

**Table 3:** Kinetic isotope effect for formic acid decomposition at 303 K

Substrate	r_H_/r_D_
HCOOD	1.3
DCOOH	3.0
DCOOD	4.4

**Fig. 12:**
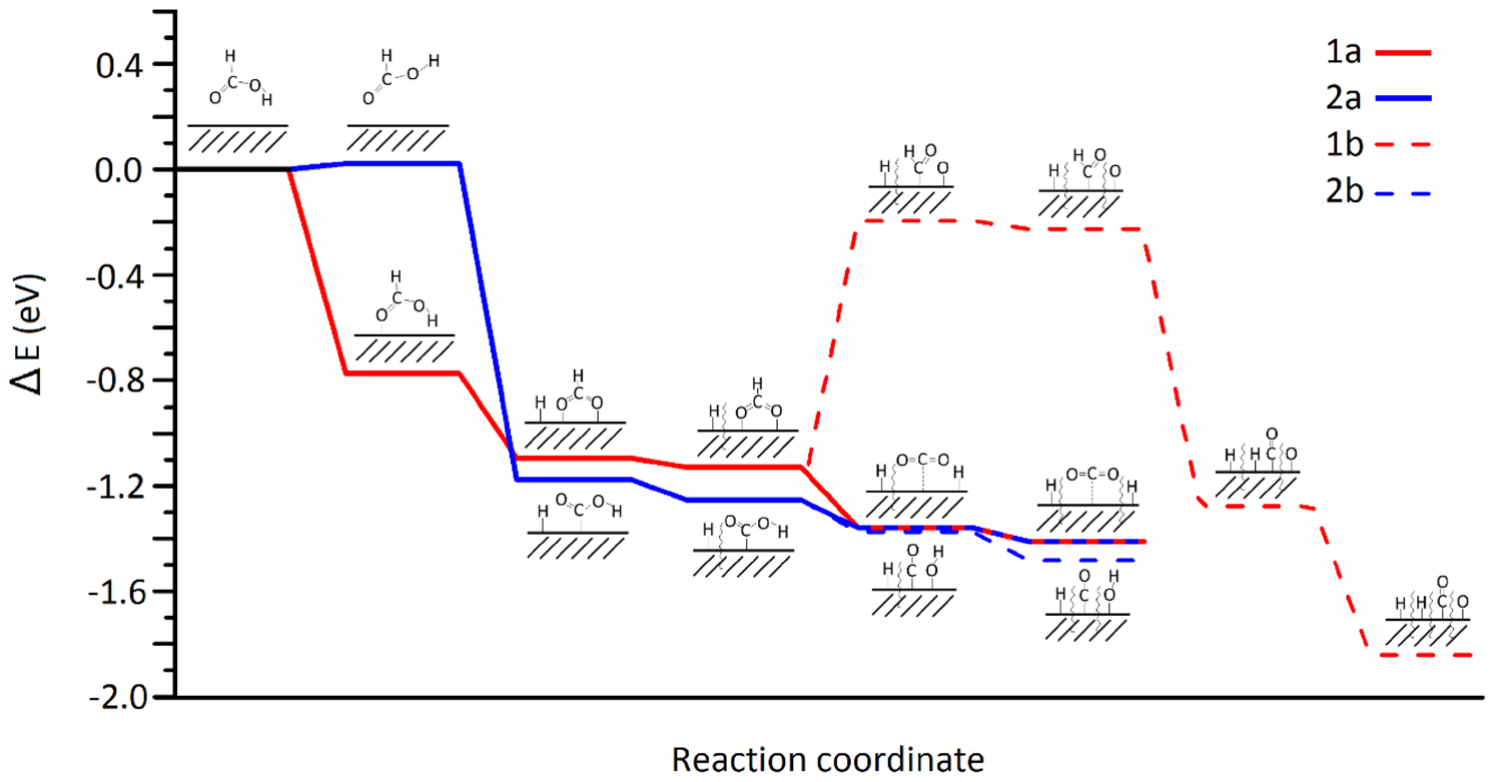
Potential energy surface for formic acid decomposition on Pd (111) surface. Red and blue lines indicate HCOO and COOH paths respectively. Solid lines lead to CO_2_ whereas dashed line, to CO

The KIE conclusions are supported by our DFT calculations. We analysed each elementary step of formic acid decomposition on Pd (111) and derived an energy profile (Fig. 12), showing the energy requirements of two different paths initiated by O–H (**1**) and C–H (**2**) dissociation. Pathway **1** begins with the adsorption of flat (trans) HCOOH (E_B_ = − 0.77 eV) and following splitting of the O–H bond (E_R_ = − 0.31 eV). The separation of the hydrogen atom and breakage of C–H suppose a further stabilisation of the system by 0.26 eV. Pathway **2** comprises a reorientation from trans configuration to cis, which is slightly more unstable than trans by 0.02 eV. After the C–H scission (E_R_ = − 1.19 eV) the hydrogen spills over the catalyst, which further stabilises the carboxylic by 0.05 eV. From this intermediate on, both pathways **1** and **2** follow the profile to produce CO_2_ (**a**), while routes **b** lead to CO formation. These results show that the formate (**1a**) and carboxylic (**2a**) intermediates are very close in energy (− 1.13 and − 1.25 eV respectively) and therefore in a competing process, susceptible to reaction barriers. However, as seen in the KIE study, HCOO is the preferable intermediate since C–H scission is largely desfavorable. Carboxylic pathway (**2a**) and following carbon monoxide route (**2b**) are as well close in energy (− 1.41 and − 1.48 eV respectively) and therefore in a competing process. On the contrary, the HCOO decomposition leads exclusively to CO_2_ and H_2_ (**1a**) because the C–O dissociation step (**1b**) is highly unfavorable (+ 0.93 eV from adsorbed HCOO). All these results support the very low ppm level concentration of CO observed experimentally. Furthermore, CO is only largely detected at high temperatures, in agreement with the KIE analysis and the thermodynamic profile. In this way, evolution of CO might be almost avoided if there are any experimental conditions that elude the COOH intermediate formation.

**Fig. 13:**
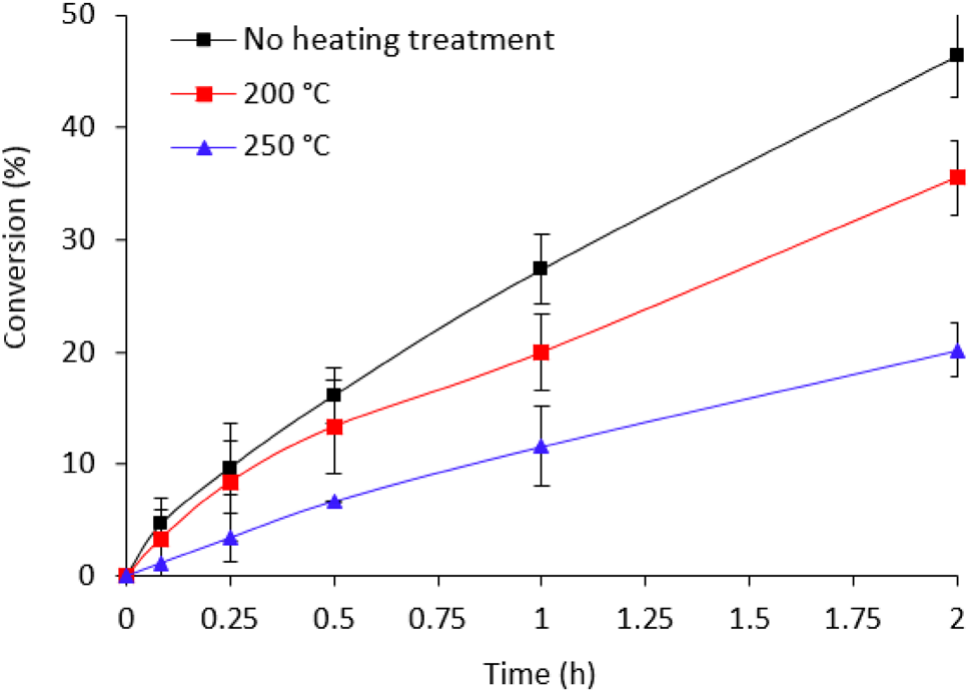
Effect of heating treatments at 200 and 250 °C. Reaction conditions: 5.3 mg of catalyst (substrate/metal molar ratio: 2000:1), 30 °C, 750 rpm, 0.5 M HCOOH, 2 h reaction time

Finally, we investigated the catalytic performance of the calcined catalysts at different temperatures. Calcination of the catalysts even at mild reaction temperature strongly affected their catalytic performance as shown in Fig. 13. When the heat treatment temperature was increased to 200 °C, we observed a decrease in initial conversion from ~ 5 to 3.5% while, at 250 °C it drastically decreased by a factor of 4. The observed catalytic trend is in agreement with the XPS and XRD data presented in Fig. 1b, c. Using Scherrer equation, we were able to calculate the mean crystallite size:

$$L=\frac{{K\lambda }}{{\beta \cos \theta }}$$

where L is the average crystallite size, λ is the X-ray wavelength in nm, β is the peak width at half maximum height in radians, K is a constant related to crystallite shape usually taken as 0.9, and θ is the peak position.

Table S1 contains the approximated crystallite size for the fresh and treated Pd/C catalysts at 200 and 250 °C. The significant increase of the Pd crystallite size during calcination process is probably due to a weak interaction between Pd particles and carbon support facilitating, thus, the agglomeration and sintering processes between particles. These and the observed XPS data of the PdO formation can explain the significant decrease of conversion shown in Fig. 13.

## Conclusions

A commercial Pd/C catalyst has been characterised and tested for the formic acid decomposition in aqueous solution as model reaction for the production of H_2_. This systematic study led to the optimal parameters of substrate/metal molar ratio of 2000:1 and 750 rpm to produce a kinetically limited reaction. In those conditions, 30 °C and a concentration of formic acid of 0.5 M, an initial TOF of 1136 h^−1^ was measured. The apparent activation energy was calculated to be 39.0 kJ mol^−1^, being this, one of the lowest values reported for HCOOH decomposition on a heterogeneous catalyst. Through catalyst characterisation techniques it was found that small nanoparticles with mean particle size of Pd below 4 nm and the presence of metallic state are the active species for the catalytic decomposition of formic acid. The results from KIE studies and computational model showed that the HCOOH dissociation follows two different paths through carboxylic and formate intermediates and while the C–H bond cleavage is a kinetically relevant step, only COOH would lead to CO poisoning of the catalyst which opens an interesting question to be solved in future research: is it possible to totally avoid the COOH pathway and thus, generate a CO-free hydrogen current?

## Electronic supplementary material

Below is the link to the electronic supplementary material.

Supplementary material 1 (DOCX 1423 KB)
