# Supplementary material for: Hydrogen Generation from Additive-Free Formic Acid Decomposition Under Mild Conditions by Pd/C: Experimental and DFT Studies
Source: Top Catal. 2018 Jan 25;61(3):254–66. doi: 10.1007/s11244-018-0894-5 (PMC6413809; doi:10.1007/s11244-018-0894-5)
Supplement: Supplementary file 1 — Supplementary material 1 (DOCX 1423 KB) [file 11244_2018_894_MOESM1_ESM.docx]

**SUPPORTING INFORMATION**

Hydrogen generation from additive-free formic acid decomposition under mild conditions by Pd/C: Experimental and DFT studies

Felipe Sanchez,^a^ Davide Motta,^a^ Alberto Roldan,^a*^ Ceri Hammond,^a*^ Alberto Villa^b^ and Nikolaos Dimitratos^a*^

***** corresponding and co-corresponding authors

*^a^Cardiff Catalysis Institute, School of Chemistry, Cardiff University, Main Building, Park Place, Cardiff, CF10 3AT, UK. E-mail: DimitratosN@Cardiff.ac.uk*

*^b^Dipartimento di Chimica, Universitá degli studi di Milano, via Golgi 19, 20133, Milano, Italy*

* corresponding and co-corresponding authors

**Calibration curves**

1. **(B)**

**Figure S1** Calibration curves of **(A)** formic acid in HPLC, and **(B)** CO_2_ in GC Varian 450.

Gas samples were analysed in a QGA-MS from Hiden Analytical to detect H_2_ and CO_2_, and Varian 450 to quantify CO_2_ and CO. In Figure S2A we display a chromatogram of the GC Varian 450 presenting the peak of CO_2_. CO is not visible in this graph due to the very low concentration of approximately 4 ppm in contrast with 44000 ppm of CO_2_ in this case. Figure S2B is a magnification of Figure S2A in order to see the CO peak with a retention time of 5.1 minutes. Figure S2C displays the MS spectra of a typical gas sample showing the identification and quantification of H_2_ and CO_2_.

**(A)**


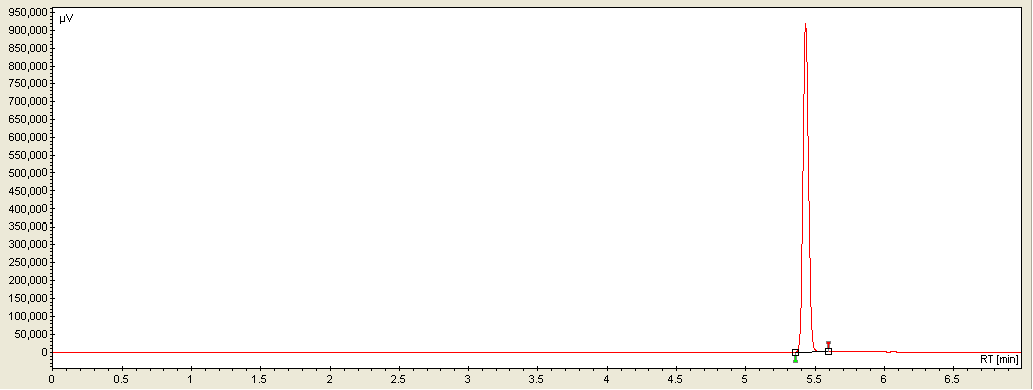


**(B)**


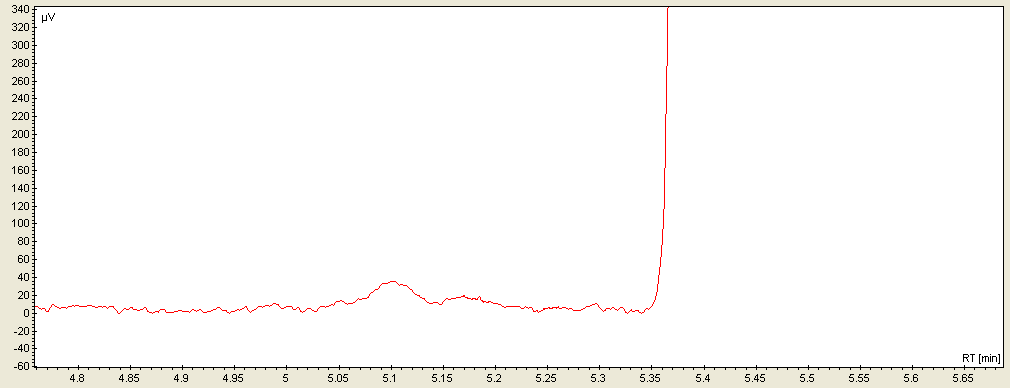


**(C)**


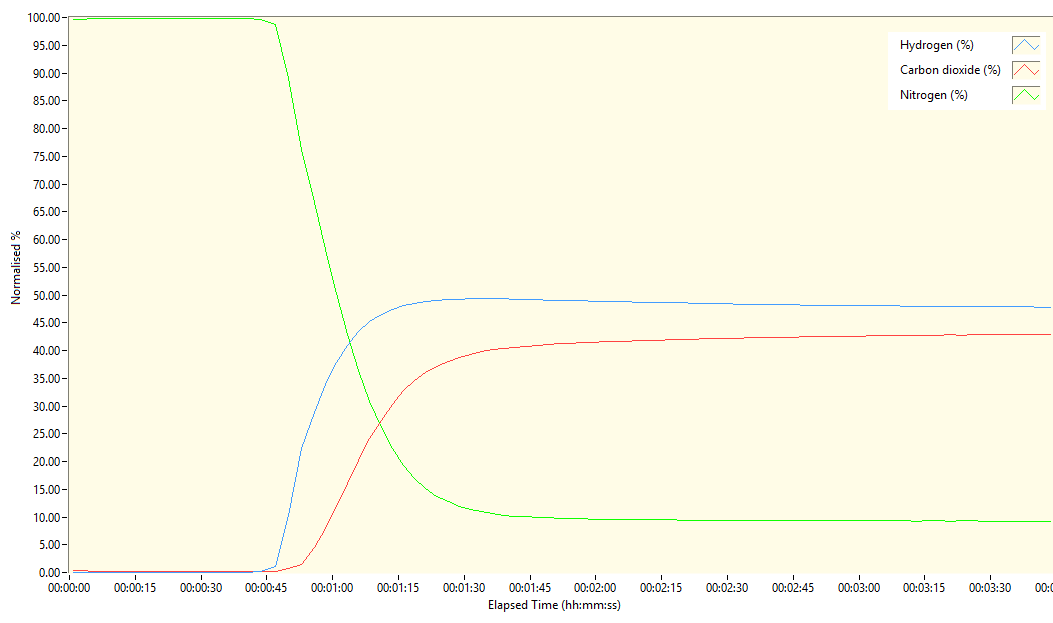


**Figure S2** Chromatogram of GC Varian 450 **(A)** CO_2_ peak, **(B)** CO peak, **(C)** MS spectra of a typical gas sample.

Figures S3A and B present the TEM images, Figures S3C and D display the particle size distribution and Figures S3E and F, HRTEM images calcined catalysts at 200 and 250 °C respectively.







D

C

B

A


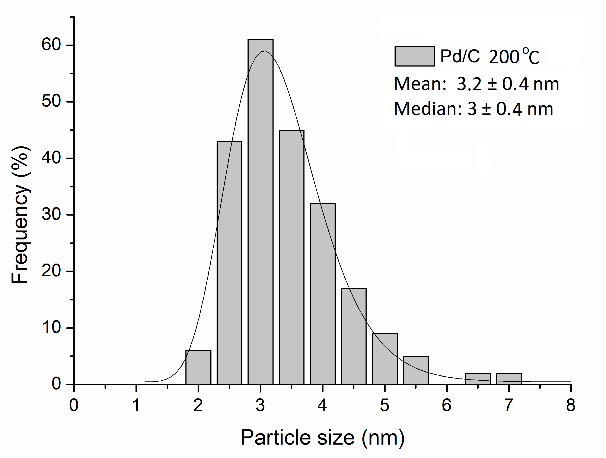

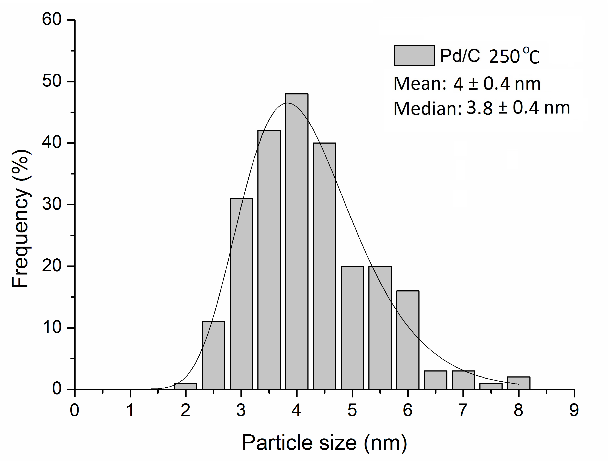


F

E


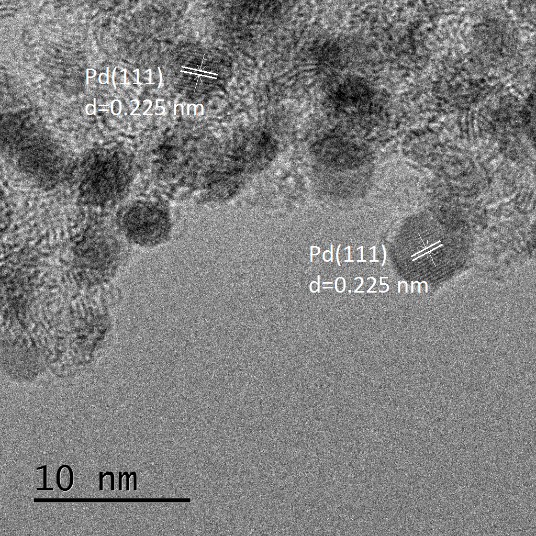

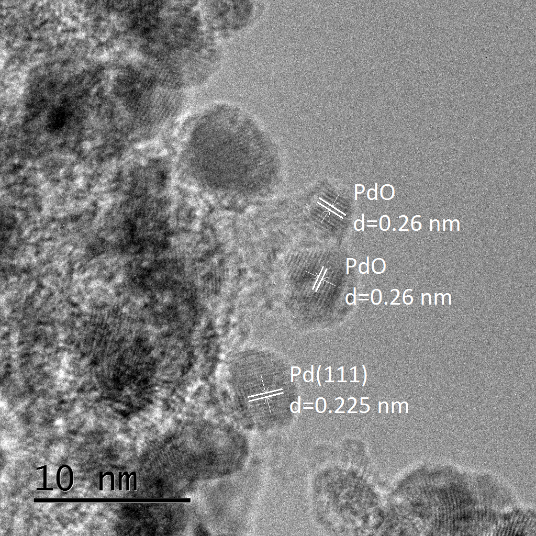


**Fig. S3** Characterisation of fresh and used 5 wt% Pd/C catalyst. (A) TEM of the fresh 5 wt% Pd/C, Magnification: 300kx, (B) TEM of the used 5 wt% Pd/C, Magnification: 250kx. Particle size distribution: (C) 5 wt% Pd/C Fresh, (D) 5 wt% Pd/C used, (E) HRTEM image of 5 wt% Pd/C Fresh calcined at 200 °C, Magnification: 600kx, (F) HRTEM image of 5 wt% Pd/C Fresh calcined at 250 °C, Magnification: 600kx.

Figure S4 displays the conversion vs time for the formic acid decomposition using the four isotopomers of formic acid.

**Figure S4** Conversion vs time for formic acid isotopomers.

| **Catalyst** | **L (nm)** |
| --- | --- |
| Fresh Pd/C | 4.78 |
| Pd/C treated at 200°C | 9.84 |
| Pd/C treated at 250°C | 10.45 |

**Table S1** Crystallite size calculated by Scherrer equation.

Figure S5 shows a QR Code for easy access to one of our Youtube videos showing one of the countless applications of hydrogen as a source of energy.

<https://www.youtube.com/watch?v=yoR8Nyo54PE&t>


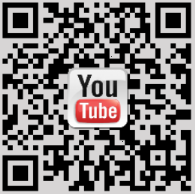


**Figure S5** QR Code for Youtube video.
